# Study of the Effect of Breast Tissue Density on Detection of Masses in Mammograms

**DOI:** 10.1155/2013/213794

**Published:** 2013-03-21

**Authors:** A. García-Manso, C. J. García-Orellana, H. M. González-Velasco, R. Gallardo-Caballero, M. Macías-Macías

**Affiliations:** Pattern Classification and Image Analysis Group, University of Extremadura, Avenida de Elvas s/n, Badajoz, 06006 Extremadura, Spain

## Abstract

One of the parameters that are usually stored for mammograms is the BI-RADS density, which gives an idea of the breast tissue composition. In this work, we study the effect of BI-RADS density in our ongoing project for developing an image-based CAD system to detect masses in mammograms. This system consists of two stages. First, a blind feature extraction is performed for regions of interest (ROIs), using Independent Component Analysis (ICA). Next, in the second stage, those features form the input vectors to a classifier, neural network, or SVM classifier. To train and test our system, the Digital Database for Screening Mammography (DDSM) was used. The results obtained show that the maximum variation in the performance of our system considering only prototypes obtained from mammograms with a concrete value of density (both for training and test) is about 7%, yielding the best values for density equal to 1, and the worst for density equal to 4, for both classifiers. Finally, with the overall results (i.e., using prototypes from mammograms with all the possible values of densities), we obtained a difference in performance that is only 2% lower than the maximum, also for both classifiers.

## 1. Introduction

 Several factors can affect the composition of breast tissue. The increase or decrease of the breast gland is part of the normal physiological changes that occur in the breast and usually occurs in both breasts simultaneously. These changes may be caused by hormonal fluctuations (natural or synthetic) including menarche, pregnancy, breastfeeding, or menopause. The increase in glandularity also depends on the woman's genetic predisposition. In young women, normally, the breast is composed mostly of glandular tissue and very little fat. And although this composition varies depending on age, it is possible to find older women with extremely dense breasts, that is, consisting mostly of glandular tissue and not fat. Weight gain or loss also increases or decreases the fat content of the breast and therefore also affects the breast glandularity [1].

The composition of breast tissue is defined by the BI-RADS parameter called “density” [2], which can have four possible values (1–4) explained in Table 1.

**Figure 1:**
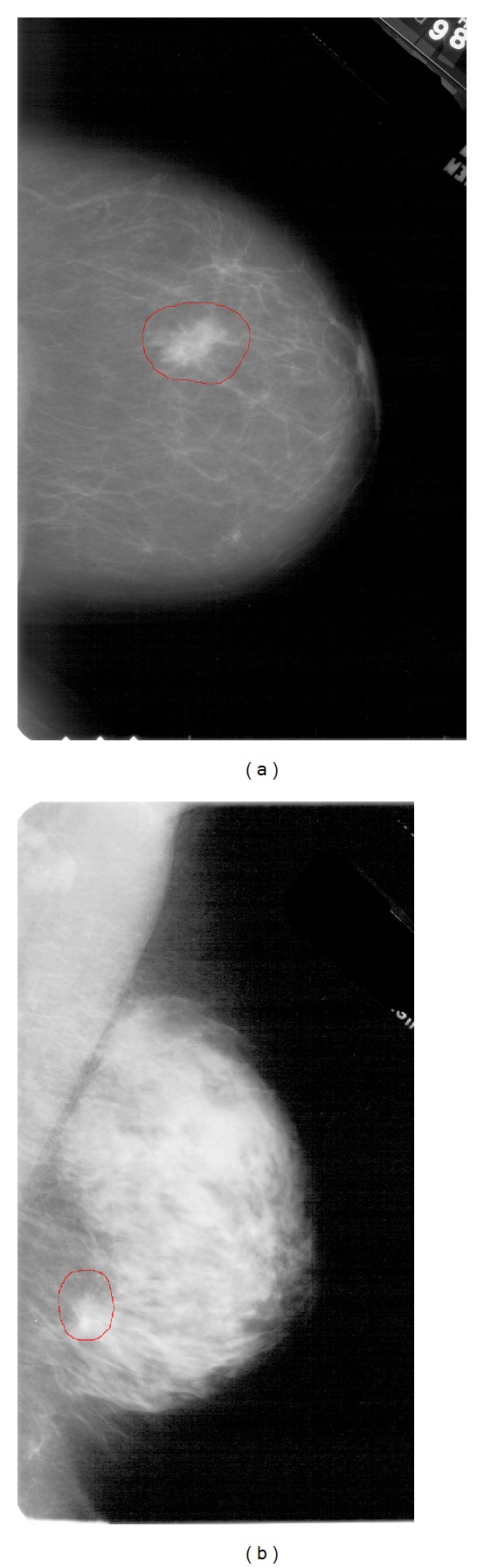
The left image shows the RCC view (right craniocaudal) of the case 1468 in USF's DDSM database that corresponds to a woman of 71 years, to which the radiologist assigned a density equal to 1. The right image shows the RMLO view (right mediolateral oblique) of the case 1985 in the same database corresponding to a woman of 41 years, and density equal to 4.

The degree of difficulty of analyzing a mammogram depends on the nature of the breast tissue, as can be seen in Figure 1. In these two mammograms, the different nature of the tissue predominant in each one is clearly distinguishable. As can be seen, it is very easy to locate the lesion in the figure on the left, which corresponds to a 71-years-old woman and has a density equal to 1, whereas it is much more difficult to analyze and locate the lesion in the mammogram on the right, corresponding to a 41-years-old woman with a density equal to 4. This example suggests that the density may be a factor limiting the sensitivity which can be reached when analyzing a mammography (both for radiologists or CAD systems). Several analyses can be found showing that the majority of cancer cases discarded in screening mammographies correspond to dense mammary gland (density equal to 3 or 4) [3–5]. We can also find in the literature works as [6], in which the impact of BI-RADS density on CAD systems is studied, in particular on the SecondLook CAD system (version 4.0) developed by the company iCAD. Finally, there are other studies such as [7] that incorporate the information provided by this parameter for the development of their algorithms to detect masses in mammograms. In this work, we studied how BI-RADS density affects our mass detection system, which consists of two stages. In the first one, a blind feature extraction is performed over ROIs, using ICA as the main technique. Next, in the second stage, those features are used as inputs to a neural classifier which determines whether the ROI includes a mass. The system is described in detail in the next two sections.

The rest of our paper is organized as follows.  Section 2 describes the general methods used for the generation of prototypes, feature extraction, and classification. Next, Section 3 includes a description of the system structure and operation, and also of the experiments devised. In Section 4, the most significant obtained results are described, while Section 5 presents the main conclusions of the work.

## 2. Methods

In this section, we present the techniques used in this study for the generation and selection of prototypes, for feature extraction tasks, and for classification. We are going to review these methods in the following subsections.

### 2.1. Data and Prototype Creation

In the literature, one can find various proposals focused on the detection and segmentation of masses on mammograms, such as those reviewed in [8], but it is usually difficult to compare the results of different studies addressing both the detection and diagnosis of masses. The main problem is the use of proprietary databases of small size, or, if using a public database, the use of selected, unspecified cases. Horsch [9] analyzes recent studies in mammography CAD and concludes that, in view of the observed variability in the datasets used, currently the only mammography database that is public and sufficiently large to allow a meaningful and reproducible evaluation of a CAD system is the Digital Database for Screening Mammography (DDSM) [10].

The DDSM is a resource available to the mammographic image analysis research community and contains a total of 2,620 cases. Each case provides four screening views: mediolateral oblique (MLO) and craniocaudal (CC) projections of left and right breasts. Therefore, the database has a total of 10,480 images. Cases are categorized in four major groups: *normal*, *cancer*, *benign,* and *benign without callback*. All cases in the DDSM were reported by experienced radiologists providing various BI-RADS parameters (density, assessment, and subtlety), BI-RADS abnormality description, and proven pathology. For each abnormality identified (within which masses are included), the radiologists draw free form digital curves defining ground truth regions. We consider these regions to define squared “regions of interest” (ROIs) that will be used as prototypes of mass. Apart from the previous data, each DDSM case includes additional information such as patient age, date of study, and digitization or digitizer's brand, though we have not used it in this work.

**Figure 2:**
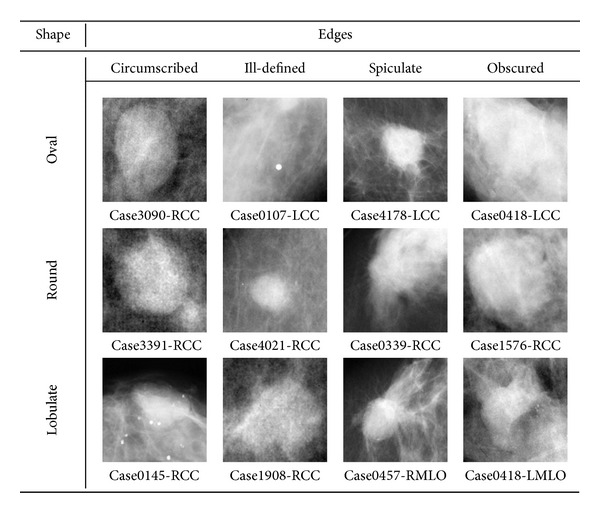
Examples of masses for each combination of shape and margins. Each ROI image has been resized to a common size of 128 × 128 pixels. Case name and view are located at the bottom of each ROI.

The DDSM database contains 2,582 images that contain an abnormality identified as mass, whether benign or malignant. Some of them were located on the border of the mammograms and could not be used (see the following paragraph, dedicated to ROIs). Consequently, only 2,324 prototypes could be considered, namely, those which might be taken centered in a square without stretching. Some mass prototype examples are shown in Figure 2.

**Figure 3:**
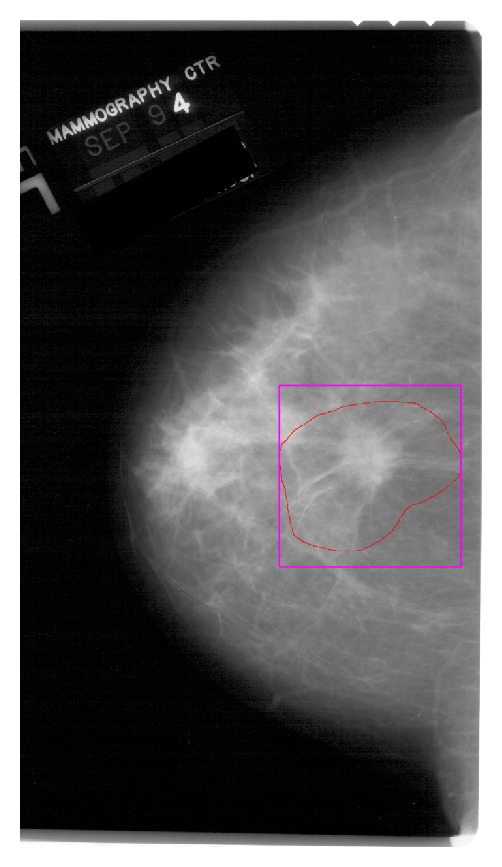
Ground truth region was defined by radiologist (red solid line) and was considered ROI (purple box) on a DDSM mammogram.

*Regions of Interest.* Ground truth regions for abnormalities are defined in the database by a chain code which generates a free hand closed curve. We use the chain code to determine the smallest square region of the mammogram that includes the manually defined region. Therefore, if the mass is located near one edge of the mammogram, this procedure may not be able to obtain a squared region from the image, and the mass is discarded as a valid prototype.  Figure 3 shows an example of the ground truth region coded by the radiologist (solid line) and the area to be used as ROI (purple box). On the other hand, the prototypes of normal tissue were selected randomly from the normal mammograms. This normal tissue prototypes were caught originally with sizes randomly ranging from the smallest to the largest of the sizes found in the DDSM for masses.

The generated regions have different sizes but the selected image feature extractor needs to operate on regions with the same size, so we need to reduce the size of the selected regions to common sizes. The reduction of ROIs to a common size has demonstrated to preserve mass malignancy information [11–13]. To determine the optimum region size, we considered two sizes for the experiments: 32 × 32, 64 × 64 pixels. The process of resizing was carried out using the bilinear interpolation algorithm provided by the OpenCV library [14].

### 2.2. Feature Extraction

**Figure 4:**
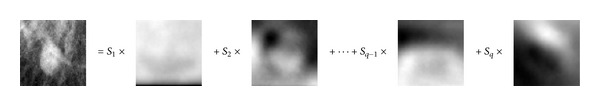
Decomposition of the image using an ICA basis.

As we commented above, we used Independent Component Analysis (ICA) [15] as blind feature extraction method. The objective of the method is to obtain an appropriate functions basis, derived from prototype ROIs (including masses and normal tissue), so that we can represent the texture and characteristics of each ROI from the breast images as an expansion in this basis (Figure 4), where the coefficients of this expansion (**s**_*i*_) are the input vectors to the classifiers (i.e., the “features” describing the ROIs).

The added value of our approach, compared to other methods that use some generic functions, is that our basis should be more specific for our problem, since it is obtained using a selection of the images to be classified.

*Independent Component Analysis.* Independent Component Analysis (ICA) defines a generative model of the observed multivariate data, typically given as a sample database. In this model, it is assumed that the data are linear combinations of some unknown latent variables, and the system by which are combined is also unknown. It is assumed that the latent variables are non-Gaussian and mutually independent, and they are called independent components of the observed data. These independent components, also called sources or factors, can be determined by ICA. ICA is related to Principal Component Analysis (PCA) [16] since, before applying the ICA method itself, it is advisable to make a dimension reduction or feature extraction of the original input vectors which can be done using PCA. The data analyzed by ICA can come from many different types of fields including digital images. In many cases, the data comes from a set of parallel signals or time series, being used in this case the term “Blind Source Separation” (BSS) to define these problems.

In that sense, if we suppose that we have *n* signals, the objective is to expand the signals registered by the sensors (**x**_*i*_) as a linear combination of *n* sources (**s**_*j*_), in principle unknown as follows:

(1) $$\begin{array}{c} x_{i}=\sum_{j=1}^{n}a_{ij}s_{j}. \end{array}$$

The goal of ICA is to estimate the mixing matrix **A** = (*a*_*ij*_), in addition to the sources **s**_*j*_. One can use this technique for feature extraction since the components of **X** can be regarded as the characteristics representing the objects (patterns) [15]. 

### 2.3. Classification Algorithm

In our system, the classification algorithm has the task of learning from data. An excessively complex model will usually lead to poorly generalizable results. It is advisable to use at least two independent sets of patterns in the learning process: one for training and another for testing. In the present work, we use three independent sets of patterns: one for training, one to avoid overtraining (validation set), and another for testing [17]. For the classification, we have used Multilayer Perceptron (MLP) [18] and SVM classifiers [19]. We have chosen these two techniques because they are widely used in classification and detection of breast cancer, as can be seen in the works listed in several reviews as [9] and in [20]. Also, to do a more rigorous study as is shown in [21], we could have tested with other techniques and other quality metrics that are also widely used in classification and regression problems, although they may not be as common in works found on detection and classification of breast cancer.

#### 2.3.1. Neural Networks

We implement MLP with a single hidden layer, and a variant of the Back-Propagation algorithm termed Resilient Back-Propagation (Rprop) [22] to adjust the weights. This last is a local adaptive learning scheme performing supervised batch-learning in a multilayer perceptron which converges faster than the standard BP algorithm. The basic principle of Rprop is to eliminate the negative effect of the size of the partial derivative on the update process. As a consequence, only the sign of the derivative is considered in indicating the direction of the weight update [22]. The function library of the Stuttgart Neural Network Simulator environment [23] was used to generate and train the NN classifiers. To avoid local minimum during the training process, each setting was repeated four times, changing the initial weights in the net at random. Furthermore, the number of neurons in the hidden layer was allowed to vary between 50 and 650 in steps of 50.

#### 2.3.2. Support Vector Machines

As with MLP, the goal of using an SVM is to find a model (based on the training prototypes) which is able to predict the class membership of the test subset's prototypes based on the value of their characteristics. Given a labeled training set of the form (**x**_*i*_, **y**_*i*_), *i* = 1,…, *l* where **x**_*i*_ ∈ *ℜ* ^*n*^ and **y** ∈ {1,−1}^*l*^, the SVM algorithm involves solving the following optimization problem:

(2) min⁡w∈ℜd,b,ξi∈ℜ+ ||w||2+C∑i=1lξisubject  to yi(wTϕ(xi)+b)≥1−ξi,             ξi≥0.

In this algorithm, the training vectors **x**_*i*_ are projected onto a higher-dimensional space than the original. The final dimension of this space depends on the complexity of the input space. Then the SVM finds a linear separation in terms of a hyperplane with a maximal (and hence optimal) margin of separation between classes in this higher dimensional space.

In the model, *C* (*C* > 0) is a regularization or penalty parameter to control the error, *d* is the final dimension of the projection space, **w** is the normal to the hyperplane (also known as the weights vector), and *b* is the bias. The parameter *ξ* is introduced to allow the algorithm a degree of flexibility in fitting the data, and *K*(**x**_*i*_, **x**_*j*_) ≡ *ϕ*(**x**_*i*_)^*T*^ *ϕ*(**x**_*j*_) is a kernel function to project the input data onto to a higher dimensional space. We used the LibSVM [24] library with a radial basis function (RBF: *K*(*x*_*i*_, *x*_*j*_) = exp⁡(−*γ*||*x*_*i*_−*x*_*j*_||^2^), *γ* > 0) as kernel function. To find the optimal configuration of the parameters in the algorithm, *γ* was allowed to vary like 2^−5^ < *γ* < 2^3^ in steps of 0.5 for the exponent, and the penalty parameter *C* between 2^−5^ and 2^10^ also in steps of 0.5 for the exponent.

## 3. Outline of the Process

 In this section, we provide an overview of the structure of our system, describing the main steps required to configure the system to discriminate prototypes of masses from prototypes of normal breast tissue.

### 3.1. System Description

We provide an overview of our system's structure, describing the main steps required to configure the system in order to discriminate ROIs corresponding to masses from ROIs corresponding to normal tissue. In addition, we will present the experiments devised to determine how the performance of these classifiers is affected by the breast density, that is associated with each mammography (and, therefore, with each ROI).

**Figure 5:**
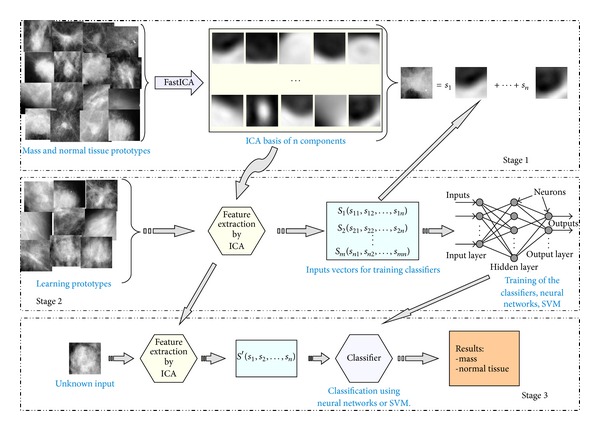
Overview of the system proposed.

The main scheme that summarizes in a more graphical form all phases of this work is represented in Figure 5. In the first stage, the prototypes of masses are obtained as was explained in Section 2.1. Then the FastICA algorithm [25, 26] is applied to obtain the ICA basis (the ICA-based feature extractor), with the *log cosh* function being used to approximate the neg-entropy. These bases are generated with different configurations, different numbers of components, and using prototypes of different sizes. The second stage uses this generated basis to obtain the training sets and to train and test the classifiers. Finally, in the third stage, the test subset, which contains input vectors not used in the optimization of the classifiers, is used to provide performance results of our system.

### 3.2. System Optimization

 To determine the optimal configuration of the system, various ICA bases were generated to extract different numbers of features (from 10 to 65 in steps of 5) from the original patches, and operating on patches of the different sizes noted above (32 × 32 and 64 × 64 pixels).

**Figure 6:**
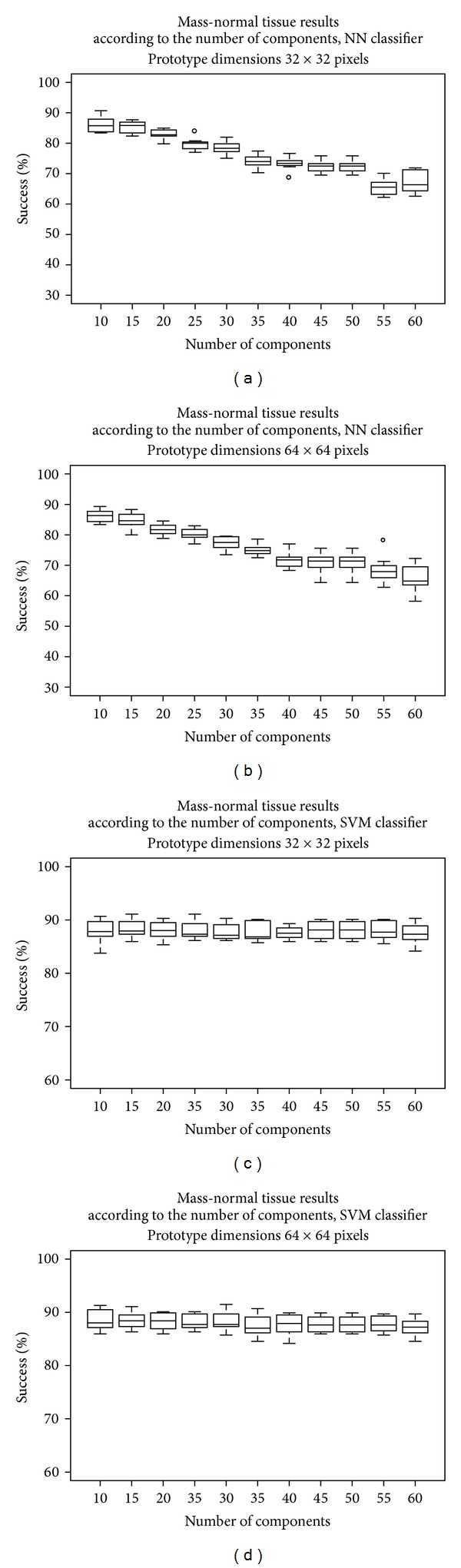
Choosing the best configuration for the feature extractor. The top row shows the results when using an NN classifier, and the bottom row shows the results for an SVM classifier. In both cases, prototypes of 32 × 32 are in the first column, and of 64 × 64 in the second column.

The training process consisted of two stages—first training the NN classifiers, and then the SVM classifiers. The results thus obtained on the test subsets in a 10-fold cross validation scheme are shown in Figure 6. This allowed us to find the optimal configuration of the feature extractor.

The study was done with a total of 5052 prototypes: 1197 of malignant masses, 1133 of benign masses, and 2722 of normal tissue.

We found that the optimal ICA-based feature extractor configuration for an NN classifier was a feature extractor that operated on prototypes of 64 × 64 pixels, extracting 10 components (average success rate 86.33%), and for an SVM classifier was a feature extractor that also operated on prototypes of 64 × 64 pixels, extracting 15 components (average success rate 88.41%). The results to be presented in the following section were obtained using these optimal configurations.

### 3.3. Experiments

To determine how the density associated to each mammography (and, therefore, to each ROI) could affect the performance of our system, we carried out five experiments. In each of the experiments we made the same tests, but with different sets of prototypes: first with all the available prototypes (one experiment), and then with prototypes obtained from mammograms with a given value of density (four experiments).

For each of the experiments, a 30-fold cross validation scheme was used. In this process, 30 partitions of the data set are generated randomly, and, iteratively, one partition is reserved for test, and the remaining 29 are used for training and validation (80% of the prototypes for training and 20% for validation). As a result we have 30 performance values that can be studied statistically.

Finally, to analyze the performance and compare results, ROC curves [27] have been generated for each experiment. To this end, the threshold applied to the output neuron of the classifier (in order to decide if the prototype being classified is mass or normal tissue) is swept, and the ratios of true and false positives are calculated. As a performance parameter, the “area under curve” (AUC) was used.

Regarding the prototypes, Table 2 shows the average number of “normal breast tissue,” “benign mass,” and “malignant mass” prototypes for each of the subsets (training, validation, and test), and calculated over the 30 “trainings of the classifier” that are made in the 30-fold cross validation scheme. These average values are shown for the overall experiment, and for the experiments with a given value of density. In the process of selection of the prototypes, no account was taken of the pathology of them. But, as can be seen, this selection process yields always a balanced distribution of the mean number of prototypes in each subset. On average, about 73% of malignant prototypes were included in the training sets, 23% in the validation sets, and 3% in the test sets. For the case of the benign prototypes, around 73% were included in the training sets, a 23% in the validation sets, and 3% in the test sets. And finally, in the case of normal prototypes, about 73% were included in the training sets, 23% in the sets of validation, and a 3% in the test sets. Therefore, if we only consider the overall data, there seems to be no clear trend which suggests that the prototypes selected in any of the ranges of density have a greater or lesser likelihood of being mass or normal tissue. However, when we analyze particular density values, differences are observed in the number of prototypes for each class that may be significant.

**Figure 7:**
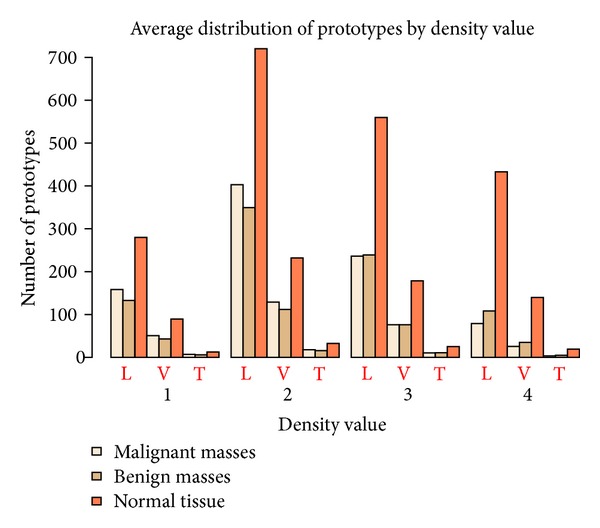
Average number of prototypes of malignant and benign masses and normal tissue divided into training (L), validation (V), and test (T) sets, distributed by density value.

In Figure 7, it can be seen that the prototypes of malignant and benign masses prototypes are quite different from the number of prototypes of normal tissue in some cases. For a density value equal to 3, this sum is always significantly lower than the number of normal tissue prototypes. For example, in the training subset this sum is equal to 475.2 and the number of normal tissue prototypes is equal to 559.6. Therefore, there is a difference of 15%. Moreover, this difference is much more significant for a density value equal to 4, where, for the training subset, the sum of malignant and benign masses is equal to 187.2 and the number of normal tissue prototypes is 432.9, being, therefore, the difference equal to 57%. In contrast, for density values equal to 1 and 2 these differences are just only a 3% and 4%, respectively, favorable to the number of mass prototypes.

## 4. Results

 As we stated above, our main interest in this paper is to evaluate the dependence presented by our system with the composition of breast tissue, determined by the BI-RADS density parameter. For this study, we have considered all those prototypes of masses in the DDSM for which a square shape could be obtained by determining the smallest squared region that includes the complete area marked by the radiologist, and always without resizing. As we commented before, the distribution of prototypes is shown in Table 2 and in Figure 7. We must point out that the relative number of prototypes of each class is very different depending on the density value. Particularly, for a density value of 4, the difference between mass (malignant and benign) prototypes and normal tissue prototypes is as high as 57%. This is a big handicap for the training of the classifiers, as we explain below.

To determine the influence of the density parameter in the performance of our system, we applied first a 30-fold cross validation scheme to train and test the system with the whole set of 5,052 prototypes. Next, a ROC analysis was performed over each of the 30 test results, calculating the area under curve (AUC) as a parameter to describe the performance over each test set. Finally, the mean value of the 30 AUCs was determined, to give a parameter that describes the overall performance of the system with those prototypes.

**Figure 8:**
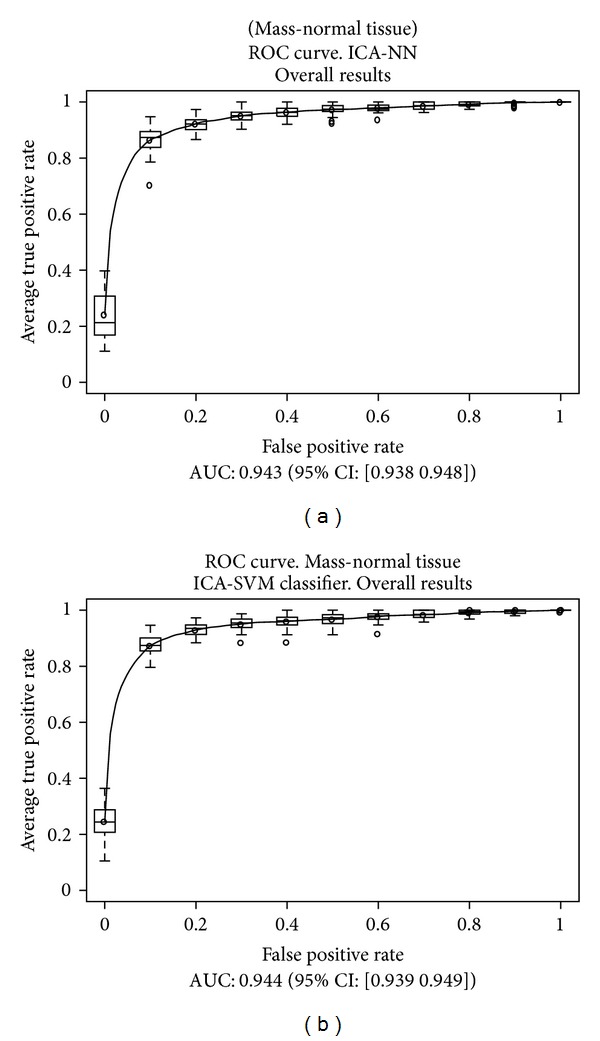
Results obtained over the test subsets, considering all the prototypes.

**Figure 9:**
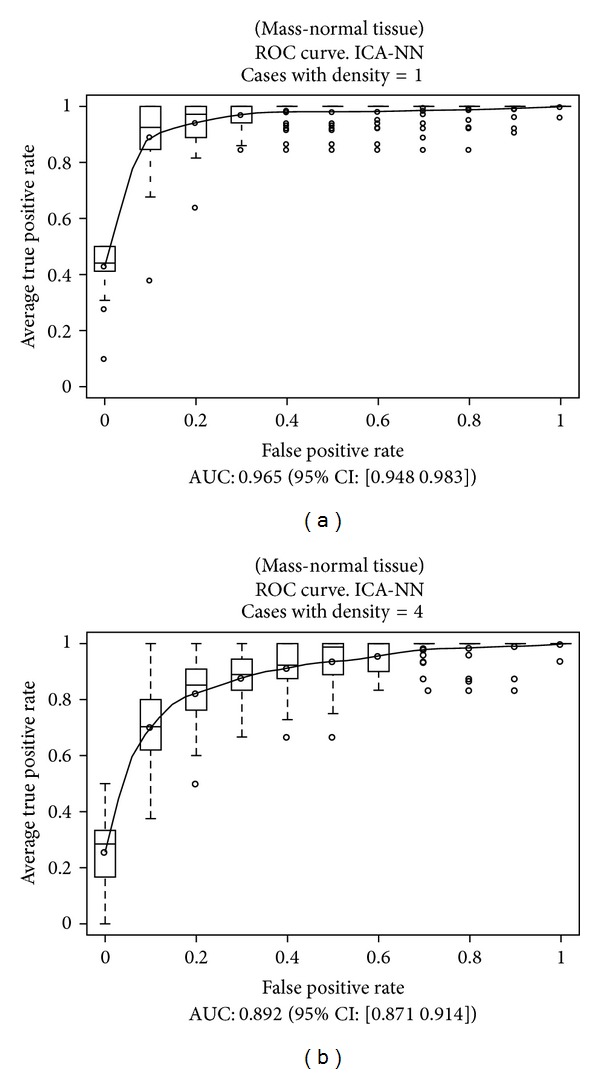
Results obtained over the test subsets, considering NN classifiers and the cases of density 1 and 4.

**Figure 10:**
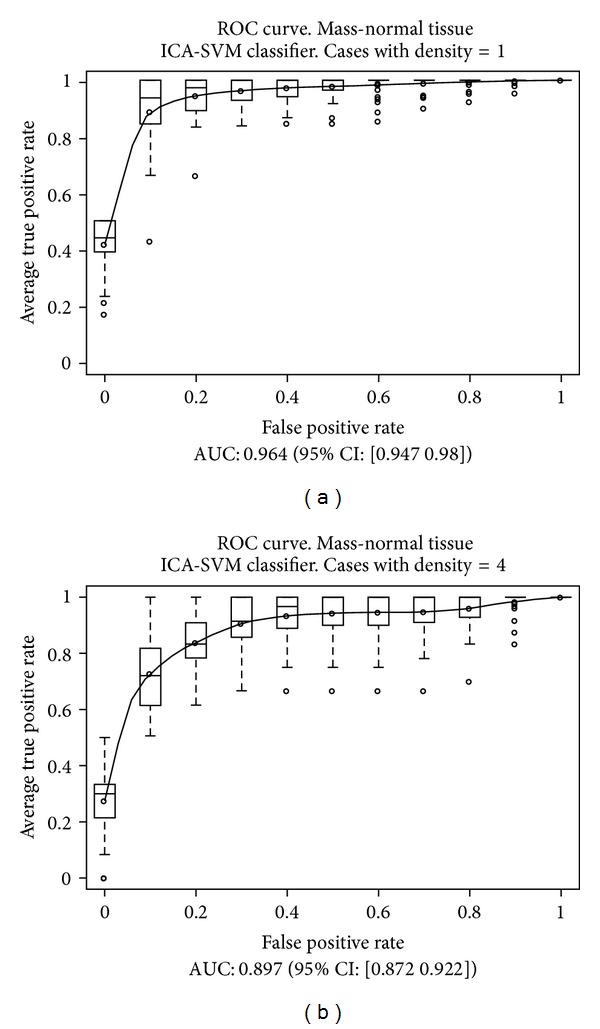
Results obtained over the test subsets, considering SVM classifiers and the cases of density 1 and 4.

This scheme was repeated later considering sets of prototypes containing only a given value of the density parameter, in order to compare the results. Those results are presented in Table 3. The overall results are presented in Figure 8 for both classifiers, and for cases with densities equal to 1 and 4 in Figure 9 for a NN classifier and Figure 10 for a SVM classifier.

As we expected, the best results were obtained for a density value equal to 1 (virtually fatty breasts with very little breast tissue, usually corresponding to old women), and the worst results for a density of 4 (very dense breasts, with much breast tissue, usually corresponding to young women). These results are consistent with other studies about the nature of cancer cases that are discarded by radiologists in a larger proportion [3–5].

Besides, it is important to remark that there are very different distributions of prototypes for the different values of density. While for a density of 1 the number of mass and normal tissue prototypes is almost the same (a 3% difference favorable to the number of mass prototypes), for a density of 4 the difference is very important (a 57% favorable to the number of normal tissue prototypes). This difference in the number of prototypes of each class introduces a statistical bias which could affect the training of the classifiers.

## 5. Conclusions

In this work, we have studied the influence of the BI-RADS density parameter assigned to a mammogram over the performance of our system. As a result, we have concluded that the performance is affected by that parameter, since the AUC of the ROC curves decreases from 0.965 to 0.892 (−7.56%) for NN classifiers and 0.964 to 0.897 (−6.95%) for SVM classifiers when we move from density 1 to density 4. However, taking into account that mammograms with density 4 are more difficult to analyze than those with density 1 (density 4 means very dense breasts with much breast tissue, so it is difficult to find masses, while density 1 means that very little breast tissue is present), and considering also the difficulties during training due to the different number of prototypes of both classes, we can conclude that our system is rather robust and performs very well even in the worst conditions.

Besides, it is important that the AUC for the global set of prototypes is only 2.28% and 2.07%, respectively, for NN and SVM classifiers, lower than the performance achieved for density 1, which is the most favourable case, so the performance of the system with the overall set is acceptable.

Finally, as the number of samples in the subsets of prototypes with densities equal to 2 and 3 is significantly higher than those in the subsets with densities equal to 1 and 4, we conclude that the variation of performance due to the BI-RADS density of our system is limited to about 7% in both cases.

On the other hand, it worth to remark the equality of performance obtained with the two types of classifiers tested.

## Figures and Tables

**Table 1:** Meaning of the BI-RADS density.

BI-RADS density	
Density value	Description
1	Breast tissue mainly fatty
2	Scattered fibroglandular densities
3	Breast tissue heterogeneously dense
4	Breast tissue extremely dense

**Table 2:** Average number of prototypes of malignant (M) and benign (B) masses and normal tissue (N) divided into training, validation, and test sets, distributed by density value.

Average distribution of prototypes by density value in the 30-fold cross validation study										
Density	Training			Validation			Test			
	M	B	N	M	B	N	M	B	N	Total
1	158.2	132.9	280.0	50.8	43.0	89.3	7.0	6.0	12.7	780.0
2	402.9	349.4	720.0	129.0	111.9	232.0	18.0	15.7	32.4	2012.0
3	236.3	238.9	559.6	76.2	76.3	178.5	10.5	10.8	25.0	1412.0
4	78.9	108.3	432.9	25.5	34.8	139.7	3.6	4.8	19.4	848.0

Overall	876.3	829.5	1192.5	281.5	266.0	639.5	39.1	37.3	89.5	5052.0

**Table 3:** This table shows the average results obtained over the different test subsets (considering all the prototypes, or only for those with a given density), as area under the ROC curve (AUC) for a confidence interval (CI) of 95%.

Mass-Normal tissue. Depending on density 30-fold cross validation test				
SVM		NN		Description
AUC	CI (95%)	AUC	CI (95%)
**0.944 **	[0.939, 0.949]	**0.943 **	[0.938, 0.948]	Overall
*0.964 *	[0.947, 0.980]	*0.965 *	[0.948, 0.983]	cases with density 1
0.959	[0.951, 0.967]	0.961	[0.954, 0.969]	cases with density 2
0.927	[0.915, 0.939]	0.916	[0.902, 0.929]	cases with density 3
***0.897 ***	[0.872, 0.922]	***0.892 ***	[0.871, 0.914]	cases with density 4
